# EHMTI-0174. Dural vessel hemodynamics during normal behavior and migraine attack

**DOI:** 10.1186/1129-2377-15-S1-A2

**Published:** 2014-09-18

**Authors:** Y Gao, P Drew

**Affiliations:** 1 Neuroscience Program, Pennsylvania State University, University Park, USA; 2 Dept. Engineering Science and Mechanics & Dept. Neurosurgery, Pennsylvania State University, University Park, USA

## Introduction

Migraine is hypothesized to be caused by the pathological dilation of blood vessels in the dura.

## Aims

To better understand the vascular pathology of migraine, we measured dural vessel diameter changes during normal behavior and under pharmacologically-induced migraine attack in awake behaving mice.

## Methods

We use two-photon laser scanning microscopy (2PLSM) to concurrently measure dural and pial surface vessel diameters down to micrometer resolution. Measurements were made in the somatosensory cortex of awake, head-fixed mice on a spherical treadmill, which allowed the mice to voluntarily run.

## Results

A majority of the dural vessels constricted during locomotion (n=29 vessels in 11 mice, peak constriction=–11.2%±5.7%), some of them did not respond (8 vessels in 4 mice), while there were a few dilated (3 vessels in 2 mice, peak dilation=12.2%±3.3%). In the contrast, all the pial vessels dilated during locomotion, with arteries showing rapid and large dilation (19.7%±8.2%) and veins showing a slower and smaller dilation (6.6%±3.2%). Preliminary experiment shows that injecting CGRP, which is believed to provoke migraine, drives the dilation of dural vessels.

## Conclusions

Dural vessels exhibit diverse dynamics during locomotion, but the dominant effect was constriction. The differences between dural and pial vessels have never been reported before, but may indicate a unique function of dural vessels during exercise. This work provides novel insights into the normal dynamics of dural vessels during behavior and their disfunction during migraine.

No conflict of interest.

